# Pickering Emulsions and Hydrophobized Films of Amphiphilic Cellulose Nanofibers Synthesized in Deep Eutectic Solvent

**DOI:** 10.1021/acs.biomac.3c00472

**Published:** 2023-08-23

**Authors:** Umair Qasim, Terhi Suopajärvi, Juho Antti Sirviö, Oskar Backman, Chunlin Xu, Henrikki Liimatainen

**Affiliations:** † Fibre and Particle Engineering Research Unit, University of Oulu, Oulu 90570, Finland; ‡ Laboratory of Natural Materials Technology, Åbo Akademi University, Turku 20500, Finland

## Abstract

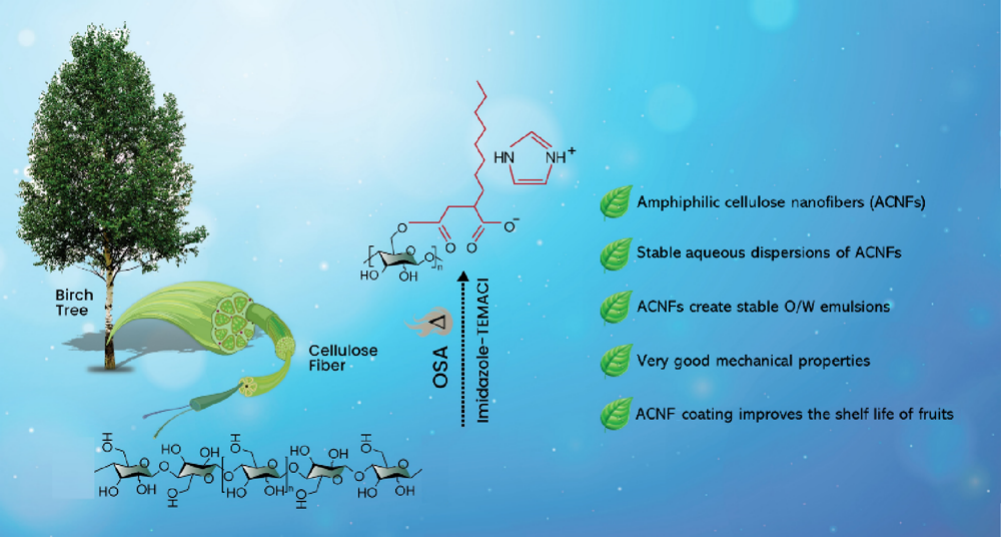

Herein, a dual-functioning deep eutectic solvent system based on triethylmethylammonium chloride and imidazole was harnessed as a swelling agent and a reaction medium for the esterification of cellulose with *n*-octyl succinic anhydride (OSA). The modified or amphiphilic cellulose nanofibers (ACNFs), synthesized using three different OSA-to-anhydroglucose unit molar ratios (0.5:1, ACNF-1; 1:1, ACNF-2; and 1.5:1, ACNF-3), were further converted into nanofibers with degree of substitution (DS) values of 0.24–0.66. The ACNFs possessed a lateral dimension of 4.24–9.22 nm and displayed surface activity due to the balance of hydrophobic and hydrophilic characteristics. The ACNFs made stable aqueous dispersions; however, the instability index of ACNF-3 (0.51) was higher than those of ACNF-1 (0.29) and ACNF-2 (0.33), which was attributed to the high DS-induced hydrophobicity, causing the instability in water. The amphiphilic nature of ACNFs promoted their performance as stabilizers in oil-in-water Pickering emulsions with average droplet sizes of 4.85 μm (ACNF-1) and 5.48 μm (ACNF-2). Self-standing films of ACNFs showed high contact angles for all the tested DS variants (97.48–114.12°), while their tensile strength was inversely related to DS values (ACNF-1: 115 MPa and ACNF-3: 49.5 MPa). Aqueous dispersions of ACNFs were also tested for coating fruits to increase their shelf life. Coatings improved their shelf life by decreasing oxygen contact and moisture loss.

## 1 Introduction

Deep eutectic solvents (DESs) are emerging green chemicals with many appealing features such as high solvent capacity, low vapor pressure, and chemical and thermal stability. They can potentially be used to eliminate negative impacts (e.g., toxicity and poor biodegradability) associated with traditional chemical reagents and solvents.^1^ DESs consist of a mixture of quaternary ammonium or metal salt and a hydrogen bond donor with a melting point significantly lower than that of the individual component.^2^ DESs derived from organic compounds have intensively been revealed in the valorization and modification of biomaterials.^3^ Particularly, they provide a sustainable and efficient medium to create functionalized cellulose nanomaterials for advanced applications.^4^

Previous studies on DES-mediated nanocellulose preparation have focused on non-derivatizing cellulose swelling prior to the mechanical liberation of cellulose nanofibers (CNFs). DESs of choline chloride, urea, or glycerol, and potassium carbonate promoted the swelling of the cell wall of cellulose fibers. Moreover, they weakened the interfibrillar interaction within the hydrogen bond network.^5,6^ The DES of oxalic acid dihydrate and choline chloride was used to hydrolyze cellulose to produce cellulose nanocrystals (CNCs) after mechanical disintegration.^7^ Some studies have reported the use of DESs as a reaction medium for synthesizing functionalized nanocelluloses.^8,9^ The DES derived from urea and lithium chloride acted as a non-degrading and non-dissolving solvent system for manufacturing succinylated CNFs.^10^ Additionally, anionic wood nanofibers were prepared in an approach using succinic anhydride in the DES based on triethylmethylammonium chloride (TEMACl) and imidazole under mild conditions (2 h and 70 °C).^11^ This DES system was also used in the esterification of CNFs and all-cellulose composite films with *n*-octyl succinic anhydride (OSA).^12^ Recently, DESs of choline chloride and carboxylic acids have been used with mechanical treatments to prepare esterified CNFs with width less than 100 nm.^13^ Similarly, an acidic DES of betaine hydrochloride and urea was used to esterify cellulose.^14^ More recently, anti-bacterial CNFs from bamboo pulp were prepared using a one-pot citric acid-choline chloride DES system, indicating that the increase in temperature and time could promote the esterification efficiency, yielding 0.19 to 0.35 mmol/g of the carboxyl group in CNFs.^15^ The dual-functioning DES (i.e., it is used as a swelling agent and reaction medium) could serve as an approach to modulate and tailor the hydrophilic–hydrophobic balance of cellulose nanomaterials for advanced applications.

Besides synthetic polymers and surfactants, nanoparticles with amphiphilic features (i.e., interfacial solid materials with affinity toward hydrophilic and hydrophobic surfaces) have been tested in numerous designs and applications including Pickering emulsions,^16,17^ films,^18^ and aerogels.^19,20^ Additionally, they are promising materials for food packaging and coatings with advanced features. As an alternative to surface-active synthetic materials, cellulose nanomaterials with amphiphilic features offer an appealing green option with minimal environmental concerns. This requires converting the inherent and dominant hydrophilicity of cellulose to amphiphilicity by the controlled grafting of hydrophobic moieties onto the cellulose backbone and the tuning of its surface charge density.^17^ Earlier functionalized biopolymers with amphiphilic properties such as chitosan have been used as fruit coatings.^21−24^ For instance, amphiphilic chitosan films were prepared by crosslinking with genipin, and the crosslinked films effectively prolonged the preservation time of strawberries.^25^ In another study, amphiphilic chitosan/carboxymethyl gellan gum composite films enriched with mustard essential oil were used to preserve mangoes for 10 days.^26^

Previously, various reaction routes have been used to fabricate nanocellulose with amphiphilic features. An acid-free, two-step reaction based on subsequent oxidation with sodium meta-periodate and reductive amination was used to obtain amphiphilic CNCs containing butylamine isomers (*n-* and *tert-*butylamine).^27^ Similarly, a two-step periodate oxidation and reductive amination resulted in benzyl-polyethylene CNCs displaying pH-responsive amphiphilicity. Recently, cellulose grafting with polyethylene glycol^28^ and aqueous counter collision^29,30^ was conducted to create amphiphilic nanocellulose. As an alternative to these conventional approaches, DESs offer a novel and sustainable approach to fabricating cellulose nanomaterials with amphiphilic character for multiple purposes.

In this study, we show a DES reaction medium based on imidazole and TEMACl for the controlled esterification of cellulose with OSA to synthesize amphiphilic CNFs (ACNFs). Particularly, we elucidate the balance between the minimum degree of substitution (DS) values and the surface charge density (SCD) values to create ACNFs-stabilized aqueous dispersions and emulsions, and self-standing films without compromising mechanical properties. Three different OSA-to-anhydroglucose unit (OSA:AGU) ratios were examined to monitor the effects of DS and SCD values on the ACNFs properties. The obtained ACNFs were characterized using transmission electron microscopy (TEM), scanning electron microscopy (SEM), Fourier transform infrared (FTIR) spectroscopy, nuclear magnetic resonance (NMR) spectroscopy, X-ray diffraction (XRD), thermogravimetric analysis (TGA), derivative thermal gravimetry (DTG), and ultraviolet–visible (UV–vis) spectroscopy. The dispersibility of ACNFs in water and the stability of oil-in-water (O/W) emulsions containing ACNFs were analyzed. In addition, selected properties such as optical, barrier, mechanical, and coating properties of self-standing hydrophobic films of ACNFs were studied.

## 2 Experimental Section

### 2.1 Materials

Kraft (*birch*) pulp (93.3% cellulose content) from UPM (Finland) containing 6.5% soluble hemicellulose and 0.2% insoluble lignin (TAPPI standard T 212 om-02 and T 222 om-02, respectively) was used as a raw material to produce ACNFs. The average pulp fiber width was 16.92 μm, and the length was 0.933 mm. Imidazole (C_3_H_4_N_2_ = 68.08 g/mol, purity >98.0%), TEMACl (C_7_H_18_ClN = 151.68 g/mol, purity >98.0%), and OSA (C_12_H_20_O_3_ = 212.29 g/mol, purity >98.0%) were purchased from TCI Europe. Ethanol (H_3_CCH_2_OH = 46.07 g/mol, purity >96%) was purchased from VWR Chemicals. Soybean oil was purchased from Sigma-Aldrich (Merck). Deionized water was used throughout this study.

### 2.2 Esterification Reaction

For the esterification of oven-dried Kraft pulp, the DES was prepared by mixing imidazole and TEMACl in a molar ratio of 7:3 (51.2 g of imidazole and 48.8 g of TEMACl) in a beaker. The DES mixture was heated to 80 °C in an oil bath under continuous stirring (100 rpm) for approximately 15 min to obtain a melted and colorless solution. Cellulose (3.15 g) was torn and added to the prepared DES mixture. After 15 min of continuous mixing, 2.0 g of OSA (0.5:1, OSA:AGU) was added to the cellulose–DES mixture and stirred (100 rpm) at 80 °C. After 2 h, the reaction was stopped by adding 100 mL of ethanol, followed by washing with ethanol (200 mL) and deionized water (200 mL) until the water ran clear. Three batches of amphiphilic cellulose were prepared using different OSA:AGU ratios, 0.5:1, 1:1, and 1.5:1. All three prepared samples (modified or amphiphilic cellulose) were stored at −17 °C before the nanofibrillation and analyses. For comparison, unmodified CNFs were also prepared using a Masuko grinder; details on preparation can be found in the Supporting Information.

The DS values of amphiphilic cellulose were measured using a titration reaction with little modification.^31,32^ Herein, 0.1 g of dried samples was dispersed in 5 mL of ethanol and a saponification of ester groups was done with 5 mL of 0.25 M sodium hydroxide (NaOH). The dispersion was left to react for 24 h. Afterward, 5 mL of water and 5 mL of 0.5 M hydrochloric acid (HCl) were added. After stirring for 30 min, the dispersion was back titrated with 0.25 M NaOH using phenolphthalein as an indicator. The percentage of ester groups was calculated using eq 1.
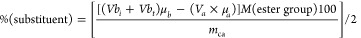
1

Here, %(substituent) is the percentage of the ester group, *Vb_i_* is the NaOH volume (L) added to the system, *Vb*_t_ is the NaOH volume (L) spent in titration, μ_*b*_ is the NaOH concentration (0.25 mol/L), *V_a_* is the HCl volume (L) added to the system, μ_*a*_ is the HCl concentration (0.5 mol/L), M (ester group) is the molar weight of the substituted group (212.29 g/mol), and *m*_ca_ is the weight (g) of the amphiphilic cellulose sample.

The DS values were calculated using eq 2.
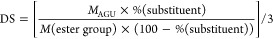
2

Here, *M*_AGU_ is the molar mass of the anhydroglucose unit (162.1406 g/mol), %(substituent) is the percentage of the substituted ester group in reacted amphiphilic cellulose (from titration), and *M*_(ester group)_ is the molar mass of the ester group (212.29 g/mol). All the measurements were conducted thrice, and average values were taken.

### 2.3 Nanofibrillation of Amphiphilic Cellulose

Before nanofibrillation, the prepared amphiphilic celluloses were diluted to a 0.5 wt % consistency and then mixed with an Ultra-Turrax mixer (IKA T-25, Germany) at 10,000 rpm for around 10 min to obtain homogenous dispersions. Subsequently, the dispersions were nanofibrillated using a microfluidizer (Microfluidics M-110EH-30, USA) in which all the samples were passed thrice through 400 and 200 μm chambers at 150 MPa. The nanofibrillated samples were analyzed for dry matter content by drying a few mg of samples in an oven at 105 °C (and also with an OHAUS MB27 Moisture Analyzer) and then stored in a cold room for further analyses. The prepared amphiphilic cellulose nanofiber samples were termed as ACNF-1, ACNF-2, and ACNF-3 (ACNFs collectively).

### 2.4 Characterization of Amphiphilic CNFs

#### 2.4.1 SCD

The SCD values of ACNFs were determined using a polyelectrolyte titration (Mütek) method. The 0.1% (w/w) ACNF dispersions and 0.5 mM buffer solutions (phosphate buffer, pH 7) were used in the analysis. The SCD values (meq/g) were calculated using eq 3.^33^
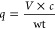
3

Here, *V* is the titrant volume (L), *c* is the titrant concentration (meq/L), wt (g) is the solid content in the sample, and *q* is the amount of charge (eq/g or meq/g). Values were measured for three repetitions of each sample and reported as an average.

#### 2.4.2 SEM

The morphological characteristics of the pristine cellulose and the prepared ACNFs were imaged using field emission scanning electron microscopy (FE-SEM, ZEISS Sigma Ultra Plus, Germany) at an accelerating voltage of 5.0 kV. For imaging, dilute aqueous dispersions of ACNFs were prepared, followed by filtration on the membranes. Before the analysis, the ACNFs-containing membranes were sputter coated with platinum (high-resolution sputter coater, Agar Scientific, United Kingdom) for 30 s at a current of 40 mA.

#### 2.4.3 TEM

To validate the conversion of pristine cellulose into nanofibers, JEOL JEM-2200FS EFTEM/STEM (Japan) was used at 100 kV. The samples for analyses were prepared following a previously reported method.^34^ After the analysis, the average widths of the individual nanofibers (analysis >30 individual nanofibers of each sample) were measured using Fiji ImageJ image processing software (version 2.6.0).

#### 2.4.4 UV−Vis Spectrometry

A UV–vis spectrometer (Shimadzu, Japan) was used to analyze the transmittance of ACNFs dispersions. The aqueous dispersions of ACNFs (0.1% w/w) were prepared, carefully poured into the quartz glass, and then placed in the spectrometer to analyze the transmittance in the 200–800 nm range against that of deionized water, which was used as a reference.

#### 2.4.5 FTIR Spectroscopy

Bruker Vertex 80 v (USA) diffuse reflectance infrared Fourier transform spectroscopy (DRIFT) was used to validate the successful esterification of cellulose. The wavelength range of analysis was 400–4000 cm^–1^, and 40 scans at a resolution of 4 cm^–1^ were used.

#### 2.4.6 NMR Spectroscopy

The ^1^H NMR spectra were recorded with a Bruker Avance 600 NMR spectrometer equipped with a 5 mm broadband (BB) probe operating at 600 MHz. About 38 mg of the sample was dissolved in 1 mL of ionic liquid, 25% tetrabutylphosphonium acetate ([P_4444_][OAc]), in DMSO-d_6_. Experiments were conducted at room temperature with 512 scans, and the positions of the peaks were referenced to the residual solvent peak.

#### 2.4.7 TGA

A Netzsch STA 449 F3 thermogravimetric analyzer (Germany) was used to study the thermal properties of the ACNFs in comparison with pristine cellulose. Approximately 5 mg of dried samples were heated in an aluminum oxide pan from 30 to 950 °C at a rate of 10 °C/min. A nitrogen environment was used at a rate of 60 mL/min.

#### 2.4.8 XRD

The crystalline structure of the pristine cellulose and ACNFs was measured through wide-angle XRD using a Rigaku SmartLab 9 kW rotating anode diffractometer (Japan) equipped with Co Kα radiation (40 kV and 200 mA). The samples were prepared using pressed tablets (thickness: 1 mm) of freeze-dried ACNFs. The spectra were obtained by scanning in a 2θ (Bragg angle) range from 2 to 50° at a scanning rate of 2°/min. The crystallinity index of the samples was calculated as follows (eq 4):^35^
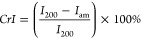
4

Here, *I*_200_ is the highest peak intensity at the (2 0 0) crystalline plane (2θ = 22.6°) and *I*_am_ is the minimum intensity at the (2 0 0) and (1 1 0) planes (2θ = 18.7°).

It should be noted that, due to the Co Kα radiation source, the cellulose peaks have different diffraction angles compared to results obtained using the Cu Kα radiation source.^7^

### 2.5 Preparation of O/W Emulsions

The soybean O/W emulsions stabilized by ACNFs were prepared using an Ultra-Turrax mixer (IKA T25, Germany) at 8000 rpm for 10 min. The oil-to-water ratio was maintained at 1:10 throughout the study. The ACNFs (0.1%, w/w) were added to the water phase before the oil was added. An O/W emulsion without ACNFs addition was used as a reference. The size of oil droplets was analyzed with a laser-diffraction particle size analyzer (LS 13 320, Beckman Coulter, USA), and three parallel measurements were performed. Droplet sizes of oil were observed and imaged using a Leica MZ LIII stereomicroscope (Leica Microsystems Ltd., Heerbrugg, Switzerland) to visualize possible aggregation after dispersion.

#### 2.5.1 Stability of O/W Emulsions

An analytical centrifuge (LUMIFuge, L.U.M. GmbH, Germany) was used to evaluate the emulsion stability at 20 °C at 3000 rpm. During centrifugation, the infrared sensor detected and measured the light transmission at 800 nm through the sample cells positioned horizontally. Hence, transmission changes could be used to indicate the formation of an O/W interface.

### 2.6 Preparation of Amphiphilic CNFs Films

Self-standing ACNFs films were prepared by vacuum filtering the ACNFs dispersions at the top of the membrane (Durapore DVPP 0.65 μm, Merck Millipore Ltd., Ireland) using a negative pressure of approximately 800 mbar. The dry matter content of ACNFs dispersions was used to design the film compositions. After the film was formed and the excess water was removed, the film was dried using a vacuum dryer (Karl Schröder KG, Germany) at 93 °C at a negative pressure of 900 mbar for 10 min. Finally, the basis weight of the film (69.3 g/m^2^) was measured.

### 2.7 Characterization of Amphiphilic CNFs Films

#### 2.7.1 SEM

The ACNFs films were imaged at an accelerating voltage of 5.0 kV using FE-SEM (ZEISS Sigma Ultra Plus, Germany). Each film was cut into small pieces and sputtered (high-resolution sputter coater, Agar Scientific, UK) with platinum for 30 s at 40 mA before analysis.

#### 2.7.2 UV–Vis Spectroscopy

The optical transmittance of the ACNFs films was measured using a UV–vis spectrometer (Shimadzu, Japan) at a wavelength ranging from 200 to 900 nm. The strips of each ACNFs film were cut and placed between the two quartz glass slides to ensure the strips were perpendicularly aligned to the incoming light beam. The transmittance from two strips of each film was measured, and an average value was reported.

#### 2.7.3 Water Contact Angle

The water contact angle was measured using Krüss DSA100 (Germany) equipment. The equipment utilized a high-speed camera (1000 frames per second) and drop analysis software. To avoid gravitational flattening, the droplet size of Milli-Q water was maintained at 2 μm. A time-domain analysis was conducted such that readings were taken every 15 s for 3 min to measure the droplet behavior on the ACNFs film surface.

#### 2.7.4 Mechanical Properties

The mechanical properties of the ACNFs films were measured using a ZwickRoell Universal testing machine (Germany). The ACNFs film samples were prepared by cutting five strips (5 mm wide and ∼60 mm long) from each film. The films were kept in a controlled environment at 50% humidity and a temperature of 23 °C ± 1 °C. The thickness of the strips was measured as an average of three random measuring points using a precision thickness gauge (England). For tensile testing, the load cell was set to 2 kN and the gauge length to 40 mm.

### 2.8 Fruit Coating Application

ACNF-1 was used as a coating for bananas to improve the shelf life through a quick dip-coating process. Fresh bananas were rinsed and dried prior to the coating analysis. The banana was dipped in ACNF-1 dispersion and placed in aluminum cups. The visual appearance of the coated and uncoated bananas was at room temperature monitored for 6 days, and their moisture loss was measured daily. The unmodified CNF was also used to coat the banana for comparison.

## 3 Results and Discussion

**Figure 1:**
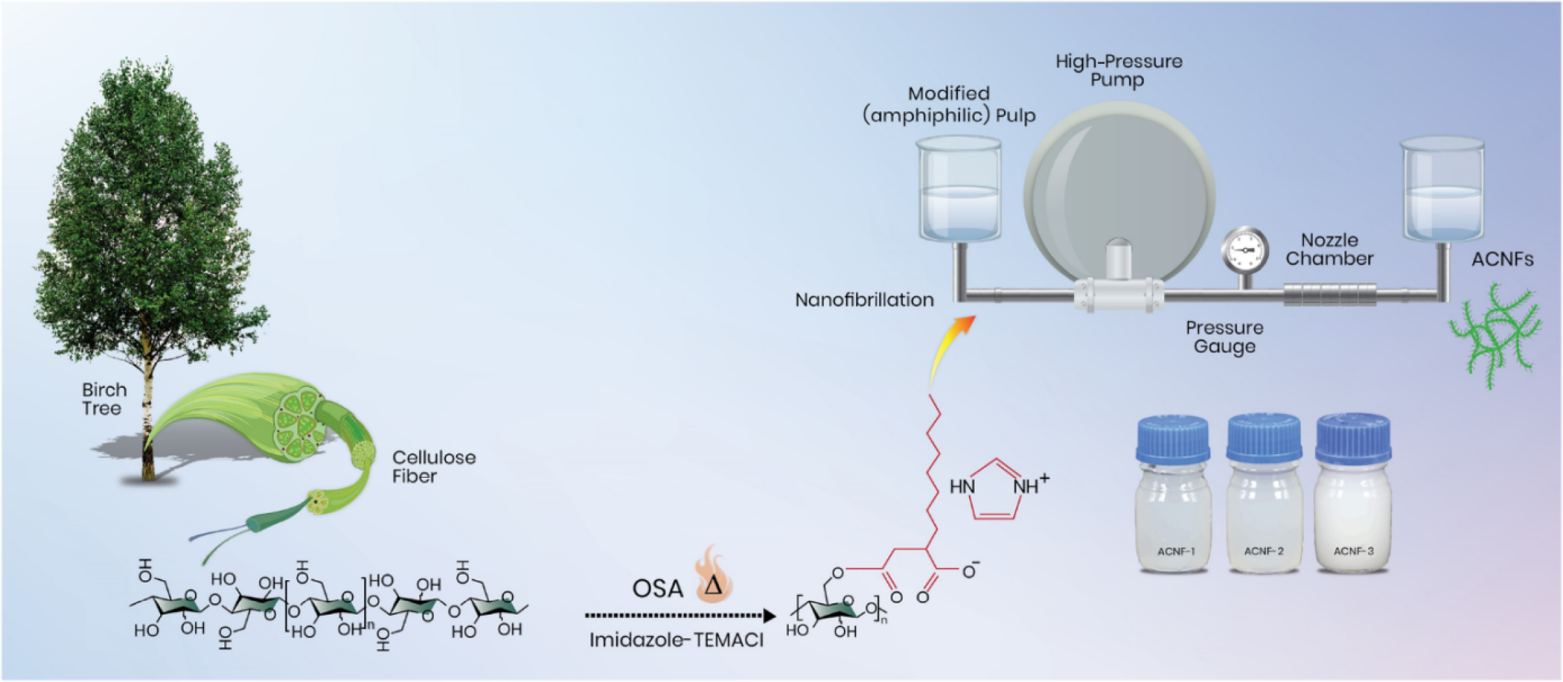
Synthesis of amphiphilic cellulose nanofibers through the esterification of cellulose pulp with *n*-octyl succinic anhydride in the deep eutectic solvent of imidazole and triethylmethylammonium chloride, followed by nanofibrillation in a microfluidizer.

A DES of imidazole and TEMACl was used as a dual-functioning medium for swelling and esterification of cellulose with OSA to fabricate cellulose with an amphiphilic character. In our earlier work, ready-made CNFs and all cellulose composite films were directly modified with OSA in the imidazole–TEMACl system. This approach was used here as a cellulose pretreatment to modulate and tailor the hydrophilic–hydrophobic balance of cellulose. Particularly, we aimed to create ACNFs-stabilized aqueous dispersions and O/W emulsions, as well as strong and hydrophobic self-standing nanocellulose films. Figure 1 shows that the esterification reaction of the anhydride groups of OSA, which was catalyzed by imidazole,^36^ introduced a long alkyl chain bearing a carboxyl group onto the cellulose.

The DES was prepared by premixing imidazole and TEMACl (molar ratio 7:3), followed by continuous heating (at 80 °C for 15 min) and mixing to get a clear, colorless liquid. Adding cellulose to the DES mixture produced a viscous dispersion with the fibers distributed evenly in the solid form without dissolution. After adding OSA, the mixture became yellowish, gel-like, and uniform in texture, indicating the successful swelling and modification of the cellulose fibers (Figure S1, Supporting Information). Ethanol was used to react with the remaining anhydrides, which eventually stopped the reaction.

The reactions were conducted using three different OSA:AGU ratios (Table 1). Based on our previous work with hydrophobized films,^12^ a low dosage of OSA was used to obtain cellulose with a low DS value. A potential amphiphilic character is attributed to the balance of hydrophilic (hydroxyl groups and carboxyl groups) and hydrophobic (long alkyl chain) surface functionalities. Previously, a higher DS value of OSA-esterified cellulose resulted in unstable dispersions and the cellulose fibers floating on the surface of the water. The cellulose mass increased (102.85, 117.46, and 142.54% for OSA:AGU ratios of 0.5:1, 1:1, and 1.5:1, respectively) after the esterification reactions, indicating that the successful modification of cellulose and imidazole–TEMACl system has an insignificant effect on the dissolution of cellulose.

**Table 1:** Degree of Substitution of Amphiphilic Cellulose, Surface Charge Density of Amphiphilic Cellulose Nanofibers, and Average Diameter of Amphiphilic Cellulose Nanofibers

OSA:AGU	sample	degree of substitution	surface charge density (meq/g) at pH 7	average diameter (nm)
0.5:1	ACNF-1	0.24	0.011	4.24 ± 0.80
1:1	ACNF-2	0.35	0.017	5.00 ± 1.59
1.5:1	ACNF-3	0.66	0.049	9.22 ± 4.51

### 3.1 Morphology

**Figure 2:**
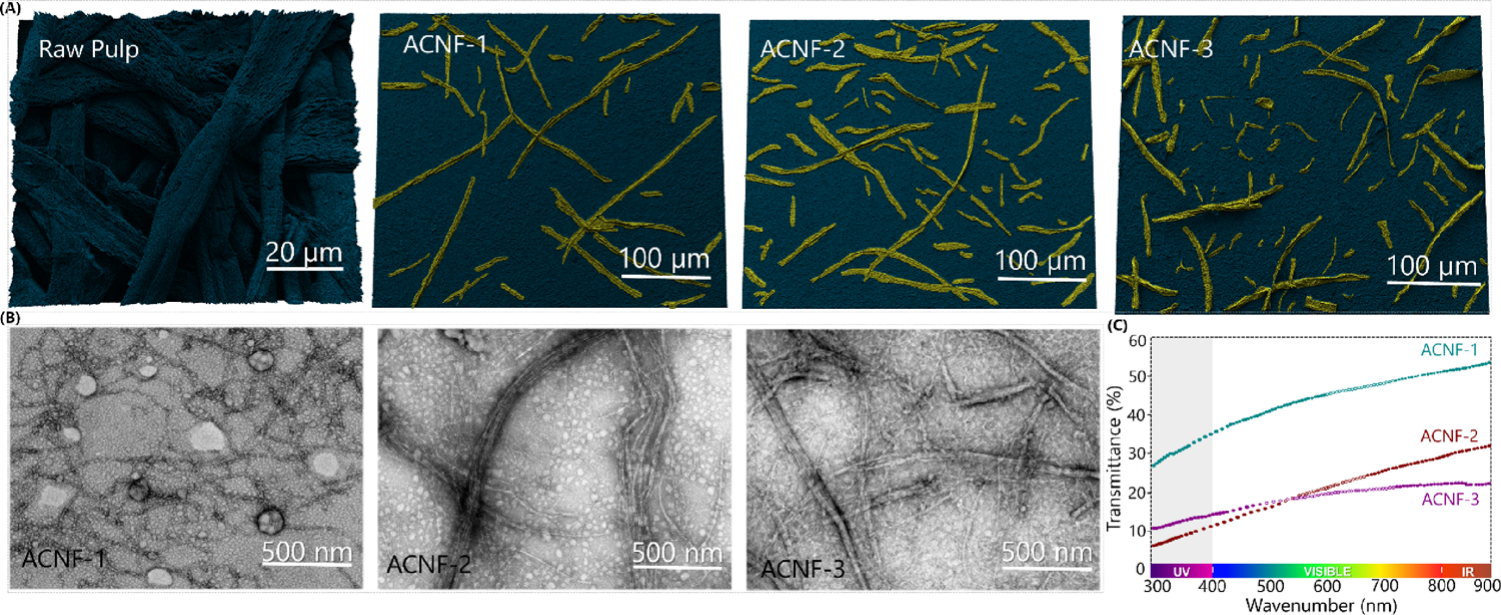
(A) Scanning electron microscopic images of pristine cellulose fibers and amphiphilic cellulose nanofibers. (B) Transmission electron microscopic images of amphiphilic cellulose nanofibers showing the successful conversion of entangled micron-sized fibers into thin nanofibers. (C) Optical transmittance of an aqueous dispersion (0.1% w/w) of amphiphilic cellulose nanofibers.

After the esterification reaction, the amphiphilic cellulose fibers were disintegrated into nanofibers by passing them thrice through the microfluidizer chambers under a pressure of 150 MPa. Figure 2 shows the morphology of the pristine cellulose fibers and obtained nanofibers (Figure S2, Supporting Information). The ACNFs had a typical elongated and flexible appearance, a heterogeneous size distribution (Figure S3, Supporting Information), and average diameters ranging from 4.24 to 9.22 nm (Table 1). Furthermore, some larger fiber fragments and nanofiber aggregates were observed in the SEM images, indicating that the cell structure of the original fibers was not completely disintegrated. Presumably, the anionic SCD (associated with the carboxylate groups contributed by the succinyl residues) was not sufficient to facilitate the swelling and weakening of the hydrogen-bonded network of fibers and enable the liberation of evenly sized nanofibers. These large fibers and aggregates significantly affected the optical properties of the ACNFs dispersions. Visibly, all three samples (ACNF-1, ACNF-2, and ACNF-3) were translucent; ACNF-1 showed a maximum transmittance of 52%, while ACNF-2 and ACNF-3 had a transmittance of 27 and 15%, respectively (Figure 2B). It is likely that the more hydrophobic ACNF-2 and ACNF-3 led to a lower degree of cellulose swelling and, therefore, ineffective nanofibrillation.

### 3.2 Characterizations

**Figure 3:**
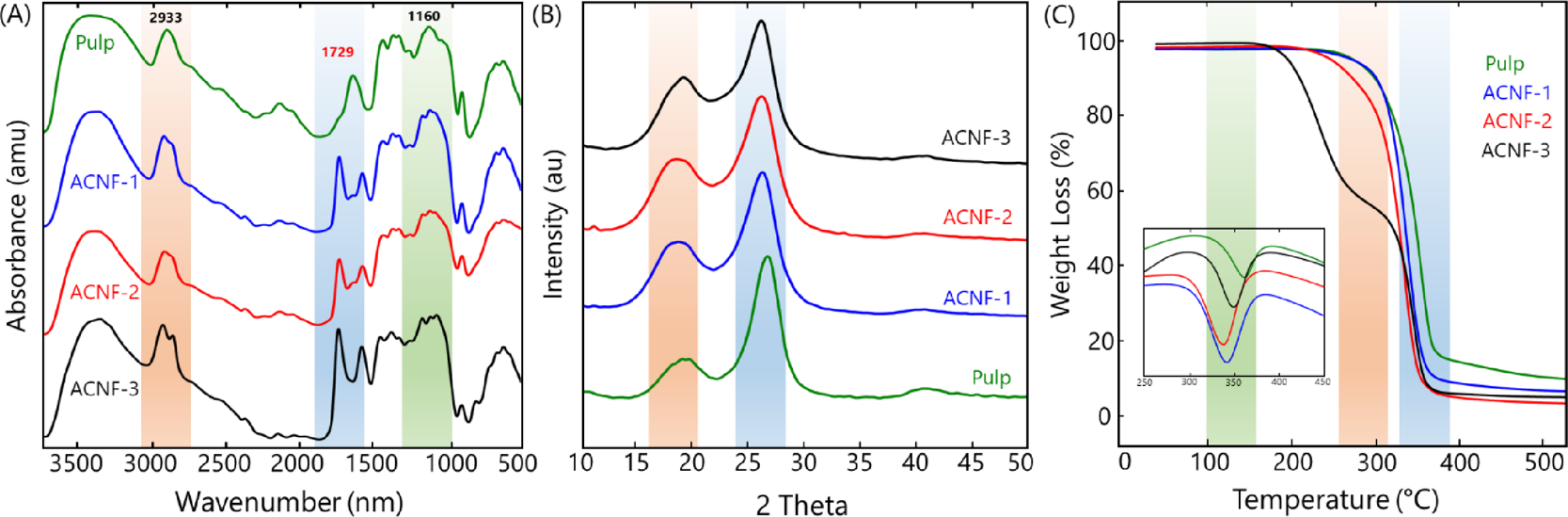
(A) FTIR spectra, (B) XRD diffractogram, and (C) TGA/DTG analysis of the pristine pulp and amphiphilic cellulose nanofibers.

FTIR analysis confirmed the successful esterification of cellulose, as shown in Figure 3A. Compared to the pristine cellulose, new peaks at 1729 and 2933 cm^–1^ existed after the esterification reaction corresponded to the C=O bond of the ester and the C–H bond of the alkyl chain (CH_3_ and CH_2_ groups), respectively (Figure S4, Supporting Information). Due to the esterification reaction of the cellulose with OSA, the number of carboxyl groups in the cellulose chain increased such that the ring opening of the OSA anhydride group resulted in the attachment of a long alkyl chain.^12^ In addition, the peak at 1160 cm^–1^ was assigned to the C–O stretching band of the newly formed ester bond^37^ while the other signals showed typical characteristics of cellulose spectra without any added functionalities.^38^

The impact of esterification on the crystalline structure of cellulose was elucidated by XRD. Figure 3B shows that the X-ray diffractograms of the ACNFs displayed a typical pattern associated with cellulose I allomorph with the peaks at 2θ = ∼19 and ∼26° attributed to the 101 and 002 diffraction planes, respectively (typical cellulose peaks shifted from 2θ = ∼19.3 and ∼22.5° to ∼19 and ∼26° due to the use of the Co Kα radiation source instead of the Cu Kα radiation source).^7^ However, a decrease in crystallinity was observed, with the crystallinity of cellulose decreasing from 76% (pristine cellulose) to 50% (ACNF-3). The role of esterification on the crystalline structure was likely minor due to the minimal DS values. This reduction was mainly induced by the intensive shear forces during the nanofibrillation of amphiphilic cellulose, which caused mechanical failure in the crystalline structure. Similar results have also been reported for other modified celluloses.^39−41^

The thermal behavior of pristine cellulose and ACNFs was examined using TGA and DTG (Figure 3C). A typical small weight loss was noticed below 100 °C, attributed to water evaporation.^39^ Cellulose decomposition started around 285 °C and above 300 °C; the primary degradation of cellulose occurred, where hydrogen bonds were cleaved.^42^ The *T*_max_ of the original cellulose was 371 °C, which was the maximum value among the samples (Table 2). The ACNFs (ACNF-1, ACNF-2, and ACNF-3) were limitedly stable and started decomposing at 355–362 °C. The decomposition temperature decreased inversely with increasing DS values. The reduction in stability was presumably due to the presence of an ester bond that is easily cleaved and the acidity of the carboxyl group, which catalyzed the degradation and the decreased crystallinity of ACNFs.^13^

**Table 2:** *T*_onset_ and *T*_max_ of TGA/DTG Analysis and the Crystallinity of Pristine Cellulose and Amphiphilic Cellulose Nanofibers

		thermal stability		
OSA:AGU	sample	*T*_onset_ (°C)	*T*_max_ (°C)	crystallinity (%)
	pristine cellulose	253	371	76
0.5:1	ACNF-1	294	362	62
1:1	ACNF-2	236	355	56
1:5:1	ACNF-3	174	360	50

### 3.3 Water Dispersions and O/W Emulsions Stabilized by Amphiphilic CNFs

**Figure 4:**
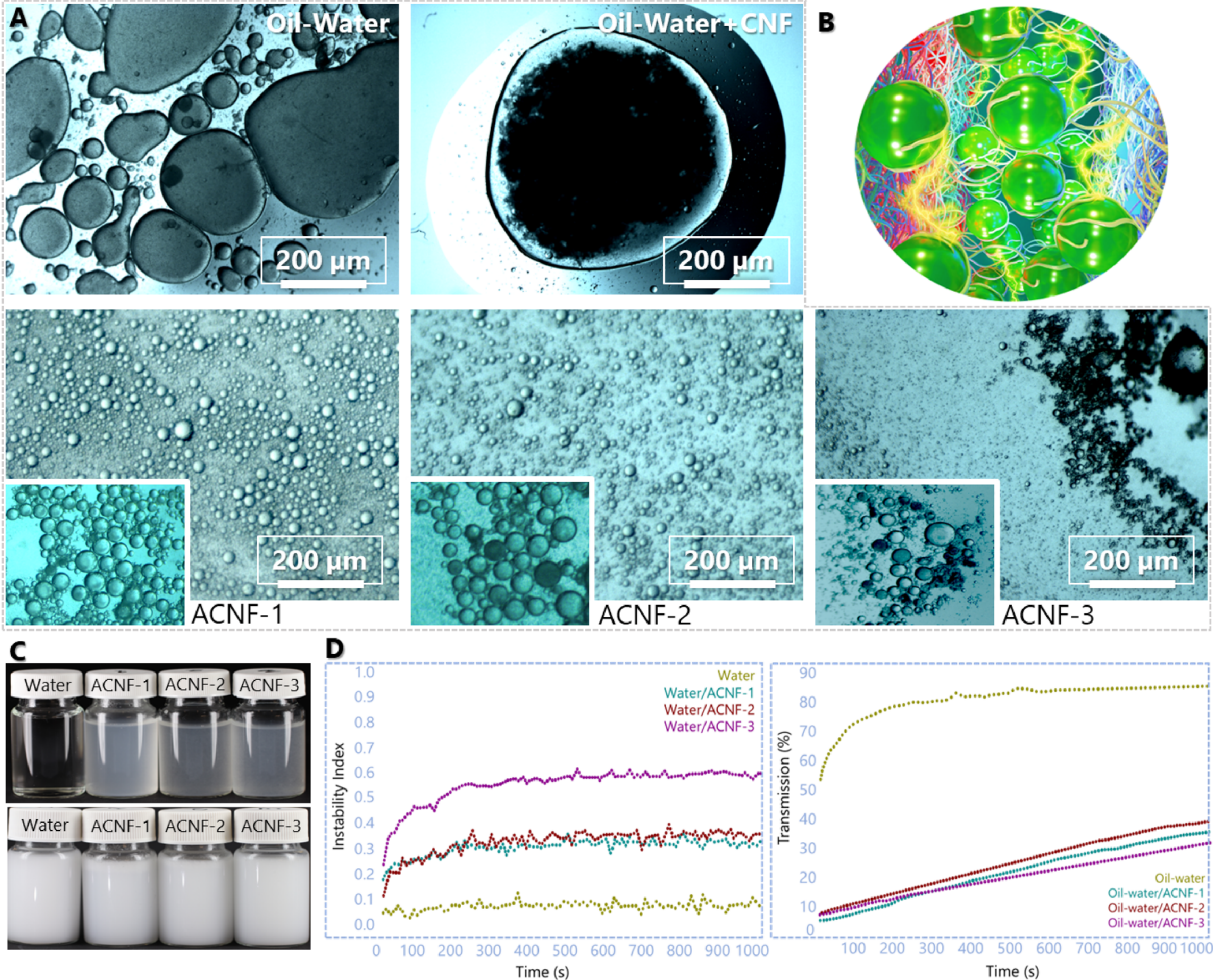
(A) The microscopy images of oil-in-water emulsions containing a reference emulsion without any stabilizer, oil-in-water emulsion with unmodified cellulose nanofibers (CNF), ACNF-1, ACNF-2, and ACNF-3. (B) Schematic illustration of oil droplets surrounded by amphiphilic cellulose nanofibers in the aqueous phase. (C) Photos of aqueous ACNFs dispersions (top) and ACNFs-stabilized soybean oil emulsions (bottom). (D) The instability index of aqueous dispersions of ACNFs. The transmission (%) of oil-in-water emulsions stabilized by ACNFs. All the measurements were conducted right after the preparation of emulsions.

The amphiphilicity of ACNFs containing long alkyl chains bearing carboxylic acid groups was demonstrated by investigating the behavior of respective aqueous dispersions and the formation of stable O/W emulsions. A laser diffraction particle size analyzer was used to determine the average oil droplet size in O/W emulsions stabilized by ACNFs (Table 3). ACNF-1 and ACNF-2 promoted a significant reduction in oil droplet size compared to O/W emulsions without any dispersant. The particle size of the reference emulsion (O/W) was 10.27 μm, the emulsion was unstable, and the separation of the oil phase occurred soon after the dispersing. Conversely, the ACNF-1- and ACNF-2-stabilized emulsions had droplet sizes (average) of 5.49 and 4.85 μm, respectively. However, ACNF-3 induced an increased droplet size of 27.56 μm, likely due to the large and aggregated ACNF-3 (Figure 4A). As shown in Figure 4A (Figure S5, Supporting Information), without ACNFs, the O/W mixture displayed large oil droplets. With the addition of unmodified nanofibers (CNF), the emulsion still appeared to be incompatible and unstable, indicating that OSA modification was a prerequisite to form a stable emulsion (Figure S5, Supporting Information). Thus, emulsions with ACNFs showed stability of oil in water with reduced droplet size, especially for ACNF-2 (4.85 μm).

**Table 3:** The Average Droplet Size of O/W Emulsions Stabilized by Amphiphilic Cellulose Nanofibers at a Concentration of 0.1 w/w %

sample	average particle size (μm)
O/W	10.27
ACNF-1	5.48
ACNF-2	4.85
ACNF-3	27.56

The stability of ACNFs aqueous dispersions (without any oil) was shown using an analytical centrifuge, which measured the light transmittance of the sample during centrifugation. All ACNFs dispersions were homogenous, opaque, and relatively stable without fast phase separation or sample floating (Figure 4C and Figure S6, Supporting Information). The stability decreased (instability index values) as a function of DS values, with ACNF-1 and ACNF-2 forming the most stable dispersions and indicating balanced hydrophilic and hydrophobic character for the aqueous medium.

Next, we investigated the function of ACNFs in stabilizing O/W Pickering emulsions. Similar to aqueous ACNFs dispersions, the stability of O/W emulsions (1:10 wt %) regarding phase separation and droplet coalescence was measured using an analytical centrifuge. The increase in transmittance is attributed to droplet coalescence and oil separation from the water phase. During the stability testing, phase separation occurred for the reference sample after 500 s of centrifugation, after which a constant high transmittance of approximately 85% was obtained. The inherent interfacial affinity of oil droplets induces coalescence, causing the formation of larger droplets and leading to phase inversion. This phase inversion can be prevented using surfactants or (nano)particles, which prevent coalescence by steric or electrostatic stabilization. Figure 4D (Figure S7, Supporting Information) shows the appearance of the O/W emulsions right after the preparation, indicating poor stability of the reference emulsion, that is, oil phase separation on the water surface. However, the emulsions with 0.1% (w/w) of ACNFs maintained stability. No phase separation was observed, as shown by the low transmittance values. The stability of emulsions containing ACNFs varied only slightly, with ACNF-3 being the most stable after 100 s of centrifugation (transmission 22%) compared to ACNF-1 (transmission 25%) and ACNF-2 (transmission 31%). The good performance of ACNFs as Pickering emulsion stabilizers was likely due to their elongated and flexible shape, which promoted steric stabilization (Figure 4B). Meanwhile, the alkyl chains bearing the carboxyl groups enhanced the interfacial adhesion of ACNFs on the droplet surface and induced stabilization.

### 3.4 Self-Standing Films of Amphiphilic CNFs

**Figure 5:**
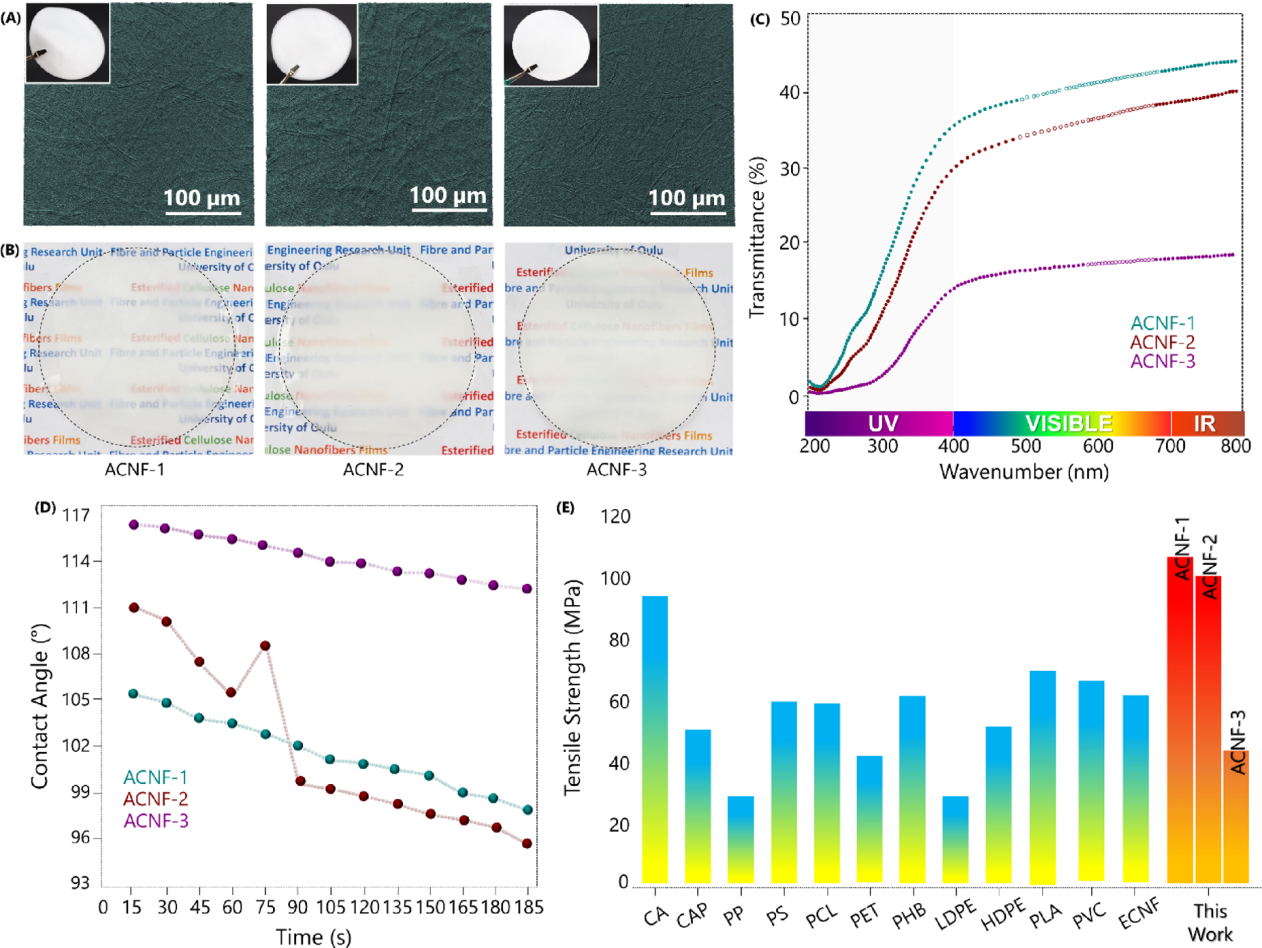
(A) Scanning electron microscopic images, (B) visual appearance, and (C) optical transmittance of amphiphilic cellulose nanofiber films. (D) The water contact angle and (E) the tensile strength of amphiphilic cellulose nanofibers and the reference films (tensile strength data of the listed materials are presented in Table S3, Supporting Information, with full name and references).

Self-standing films were prepared from aqueous dispersions of ACNFs by vacuum filtration. As shown in the SEM surface images (Figure 5A), the randomly oriented nanofiber network contained some large fiber bundles, and the films had a hazy appearance, the transmittance varying from 44% (ACNF-1) to 15% (ACNF-3). These films showed excellent foldability (Figure S8, Supporting Information). The esterification reaction significantly increased the hydrophobicity of the films. The water contact angle of the films increased linearly, at the beginning of the analysis, with the DS values from 105.2° ± 6.0° (ACNF-1) to 116.3° ± 1.43° (ACNF-3) (Figure 5D and Table S1, Supporting Information). The water contact angle was stable for around 1 min and then started to decline slightly and dropped at 97.6, 95.3, and 112.1° for ACNF-1, ACNF-2, and ACNF-3, respectively. However, the decrease in the water contact angles was not significant over time indicating that the developed ACNFs could create hydrophobic surfaces, even with the low DS values, due to the attachment of long alkyl group chains onto the nanofibers.

The mechanical properties of the ACNFs films in terms of tensile strength, tensile modulus, and elongation at break are shown in Table S2, Supporting Information. The Young’s modulus decreased from 6.92 ± 0.72 GPa (ACNF-1) to 4.89 ± 0.60 GPa (ACNF-3) with increasing DS values, suggesting decreased hydrogen bonding between the hydroxyl group of the cellulose backbone.^37^ Therefore, the ACNFs films became more ductile after introducing a long alkyl chain onto the cellulose. Similarly, the ACNF-1 film showed the maximum tensile strength of 114.92 ± 14.69 MPa, followed by ACNF-2 (106.97 ± 4.90 MPa) and ACNF-3 (49.49 MPa ± 14.37). These mechanical properties of the ACNFs films are comparable to those of previously reported CNF films and higher than many films based on synthetic or bio-based polymers (Figure 5E).

### 3.5 Amphiphilic CNFs Performance as Fruit Coatings

**Figure 6:**
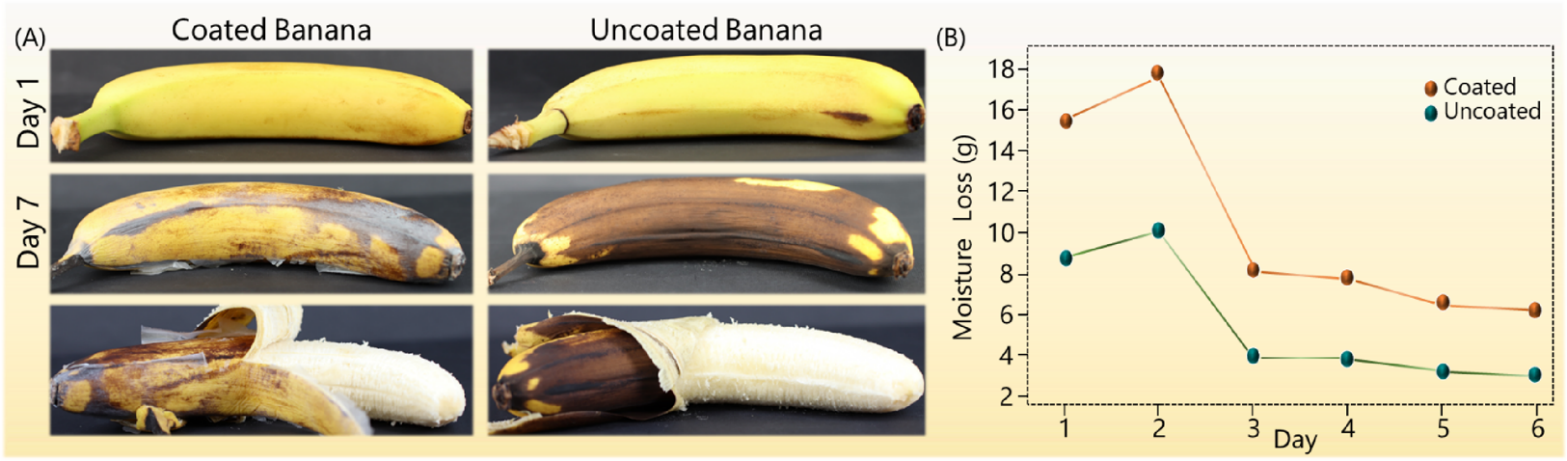
(A) Shelf life of the banana coated with amphiphilic cellulose nanofibers (ACNF-1) in comparison with the uncoated banana (visual appearance). (B) Moisture loss data for ACNF-1-coated and uncoated bananas.

ACNFs were demonstrated as coatings for fruits to improve their shelf life using a quick dip-coating process with bananas. The visual appearance and weight loss of dip-coated bananas were monitored and recorded over 6 days. The ACNFs coatings were thin, transparent, and invisible to the naked eye, indicating that the coatings do not negatively affect the appearance of the fruits. Among the three ACNFs dispersions, only ACNF-1 was further studied because of its high viscosity that enabled the creation of a uniform coating layer. After 1 week, the uncoated bananas showed significant browning on the exterior due to the accelerated cell respiration rate caused by the production of ethylene gas, which is typical for climatic fruits.^43^ Conversely, the coated banana showed little to no brown spots on the exterior, indicating that the ACNF-1 coating significantly prolonged the shelf-life and preserved the banana peel colors (Figure 6). In addition, the flesh of the coated banana showed a light-yellow color, while the non-coated banana had a darker brown color, demonstrating that the coating preserved the freshness of the banana (Figure 6). The unmodified CNF was also used as a reference coating (Figure S9, Supporting Information). Due to the high viscosity of unmodified CNF, it was easier to coat on the banana surface; however, the coating was not uniform enough to prolong the shelf-life of the banana. Just after 4 days, the banana coated with unmodified CNF showed brown spots on the exterior, and after 6 days, the exterior of the banana turned significantly dark brown compared to ACNF-1 (Figure S9, Supporting Information). The results from moisture loss data indicated no significant difference in moisture loss between coated and uncoated bananas. However, in the beginning, the moisture loss percentage is higher for coated bananas, which is attributed to the water loss from the ACNF-1 dispersion coating.

## 4 Conclusions

The DES system based on imidazole and TEMACl was an efficient, dual-functional medium for the swelling and esterification of cellulose. The modified cellulose, with an amphiphilic character, had DS values ranging from 0.24 to 0.66 and SCD values ranging from 0.011 to 0.075 meq/g. The amphiphilic cellulose was successfully nanofibrillated to produce nanofibers with an average diameter ranging from 4.24 ± 0.80 to 9.22 ± 4.51 nm. The ACNFs showed surface activity due to the balance of hydrophobic and hydrophilic characters. They promoted the stability of O/W emulsions against coalescence and reduced emulsion droplet size by adding 0.1 wt % ACNFs. Self-standing films of ACNFs showed high water contact angles ranging from 94.48° to 114.12°, their mechanical properties having an inverse relationship to DS values; however, the tensile strength of ACNF-1 (115 ± 14.69 MPa) was still comparable to some conventional plastic films. ACNFs were also used as a coating for fruits to improve their shelf life. The coated banana displayed a significant color and freshness preservation due to reduced oxygen contact and moisture loss.

## Supporting Information Available

Synthesis protocol of unmodified cellulose nanofibers; the appearance of amphiphilic cellulose nanofibers in DES; morphology of amphiphilic cellulose nanofibers (ACNFs); scanning electron microscopy, transmission electron microscopy, confocal laser scanning microscopic images; diameter distribution curves of ACNFs based on transmission electron microscopy images; ^1^H nuclear magnetic resonance spectra of amphiphilic cellulose nanofibers; oil-in-water emulsion in the presence of unmodified cellulose nanofibers; light transmission (%) indicating the stability of water and the aqueous dispersion of ACNF-1, ACNF-2, and ACNF-3; light transmission (%) indicating the stability of oil-water and the oil-water emulsions stabilized by ACNF-1, ACNF-2, and ACNF-3; foldability of films of amphiphilic cellulose nanofibers; the water contact angle values of ACNFs films; tensile strength, tensile modulus, and elongation at break of films of amphiphilic cellulose nanofibers; names and references of materials; banana coated with unmodified cellulose nanofibers and monitored for 6 days (PDF)
