# Supplementary material for: Pickering Emulsions and Hydrophobized Films of Amphiphilic Cellulose Nanofibers Synthesized in Deep Eutectic Solvent
Source: Biomacromolecules. 2023 Aug 23;24(9):4113–22. doi: 10.1021/acs.biomac.3c00472 (PMC10498439; doi:10.1021/acs.biomac.3c00472)
Supplement: Supplementary file 1 — bm3c00472_si_001.pdf [file bm3c00472_si_001.pdf]

Supporting Information

Pickering Emulsions and Hydrophobized Films of Amphiphilic Cellulose Nanofibers Synthesized in Deep Eutectic Solvent

[a]Umair Qasim, [a]Terhi Suopajärvi, [a]Juho Antti Sirviö, [b]Oskar Backman, [b]Chunlin Xu, [a]Henrikki Liimatainen*

[a]Fibre and Particle Engineering Research Unit, University of Oulu, Oulu, 90570, Finland

[b]Laboratory of Natural Materials Technology, Åbo Akademi University, Turku, 20500, Finland

*Corresponding Author: Henrikki Liimatainen (henrikki.liimatainen@oulu.fi)

Preparation of Unmodified Cellulose Nanofibers

Unmodified cellulose nanofibers (CNF) were prepared using a mechanical grinding method adopted from Laitinen et al. (2020).[1] First, 100 g (abs.) birch cellulose pulp from UPM (Finland) was soaked with 2000 g of deionized water overnight. Then, using a wet disintegrator at 30,000 rpm, three parallel pulp batches were wet disintegrated in accordance with ISO 5263-1:2004. Next, 100 g of disintegrated pulp with a consistency of 1.5 wt % was ground using a Masuko supermasscolloider (MKCA6-2J, Japan). The pulp was passed through the grinder 14 times using the following grinding stone gap values: 0 µm for three times, 20 µm for three times, 40 µm for three times, 60 µm for three times, 80 µm once, and 90 µm once. Finally, 1.7 wt % nanocellulose suspension was collected and stored at 4 °C.

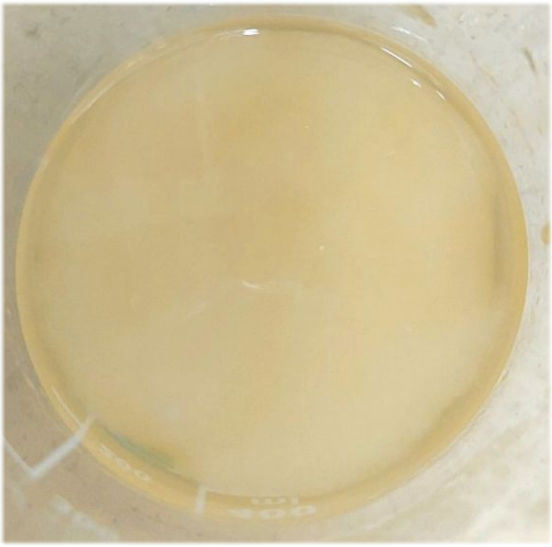

Figure S1. Amphiphilic cellulose nanofibers in the deep eutectic solvent of imidazole and triethylmethylammonium chloride in the presence of n-octyl succinic anhydride after two hours of reaction at 80 °C.

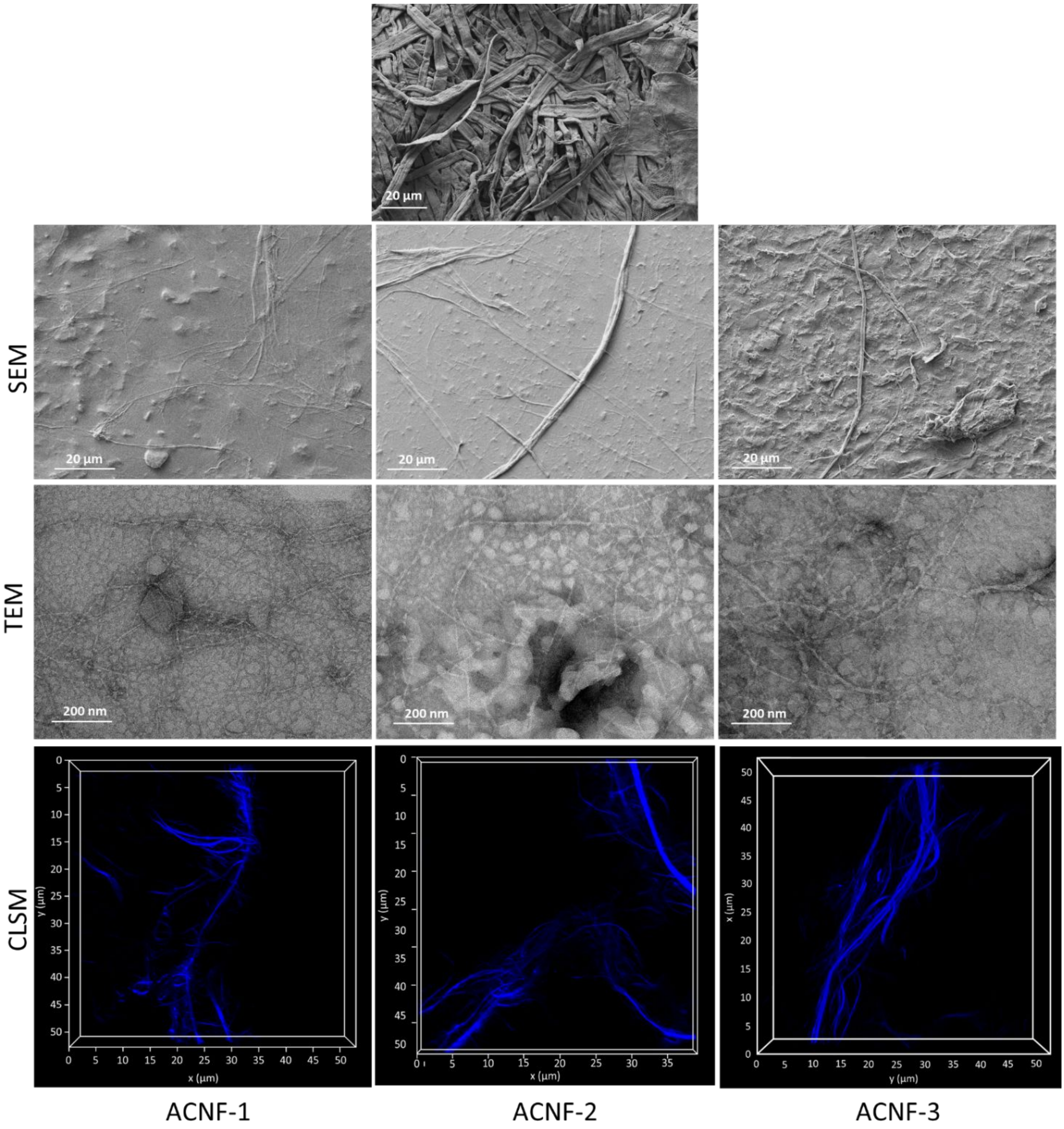

Figure S2. Scanning electron microscopy (SEM), transmission electron microscopy (TEM), and confocal laser scanning microscopy (CLSM) images of pristine cellulose (top) and amphiphilic cellulose nanofibers.

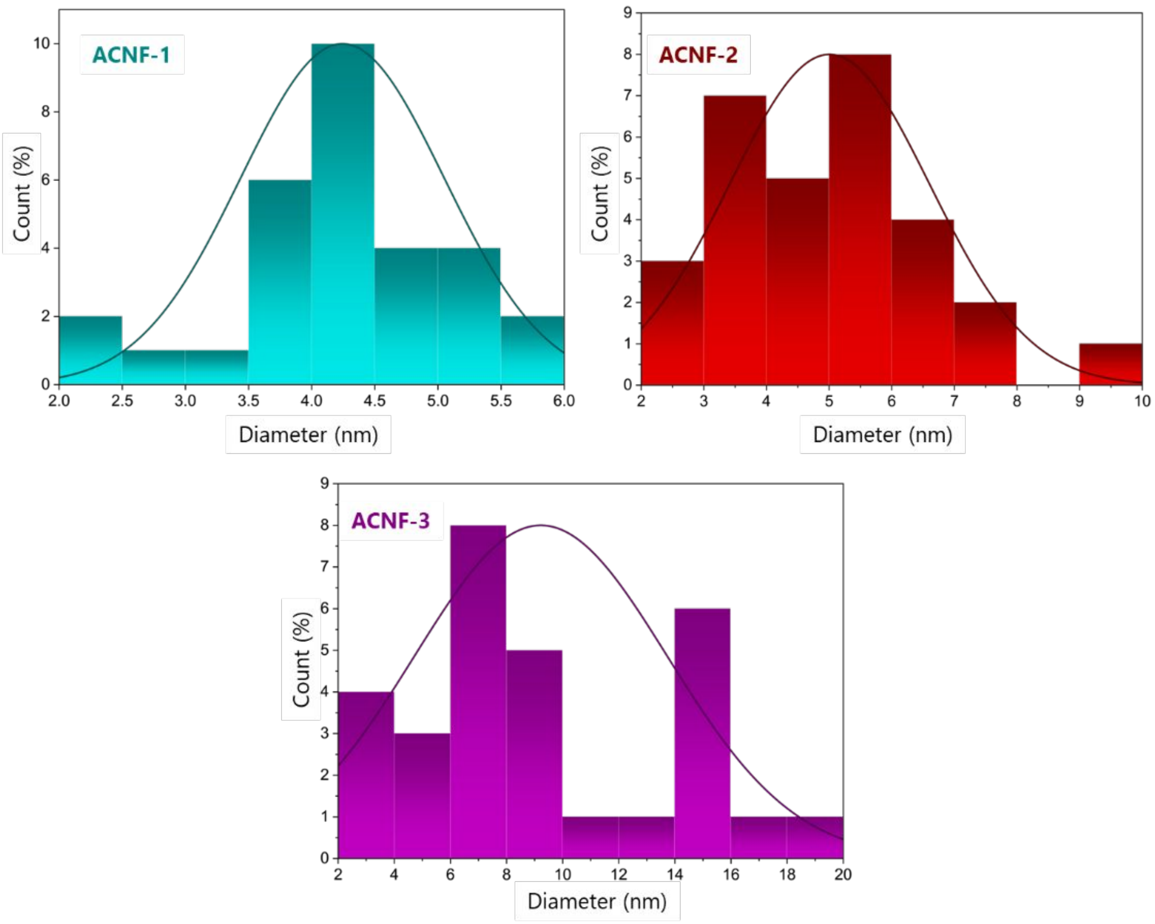

Figure S3. Diameter distribution curves of ACNFs based on transmission electron microscopy images.

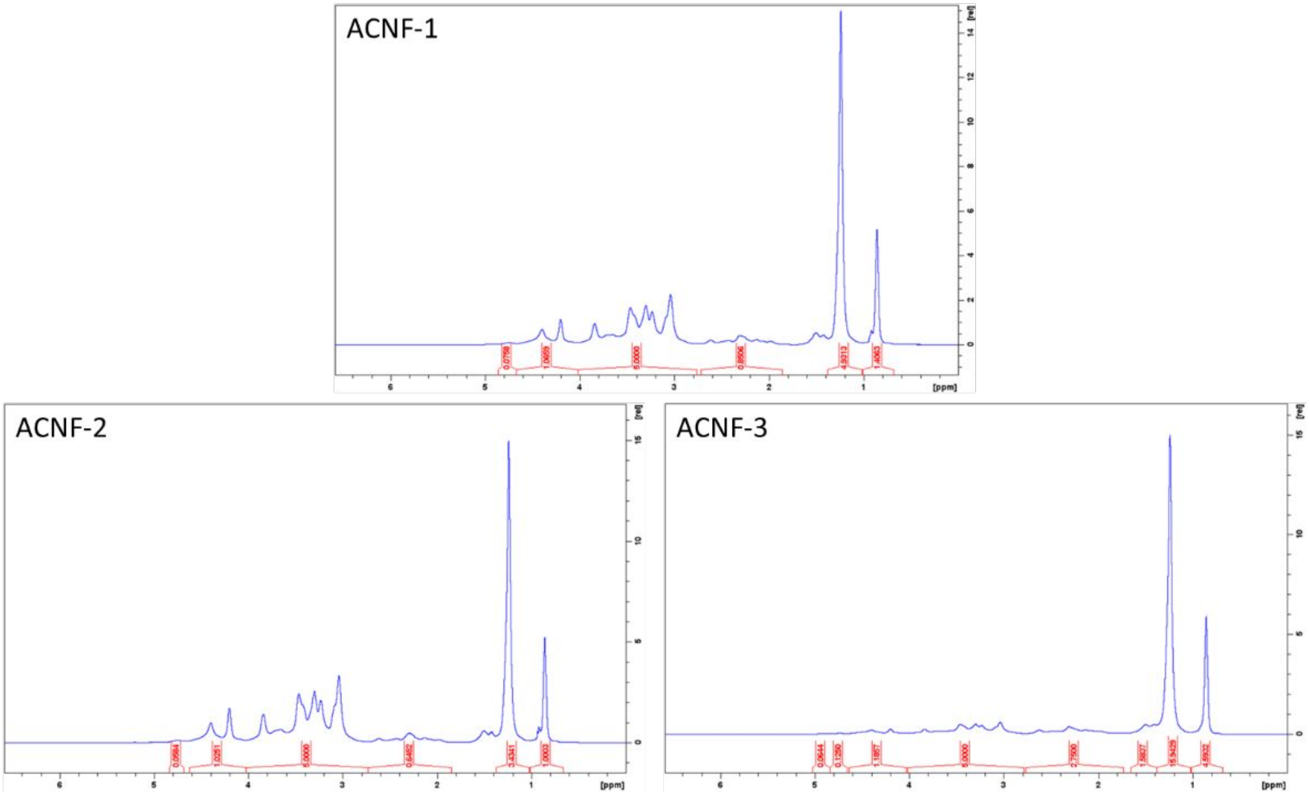

Figure S4. 1H nuclear magnetic resonance spectra of amphiphilic cellulose nanofibers.

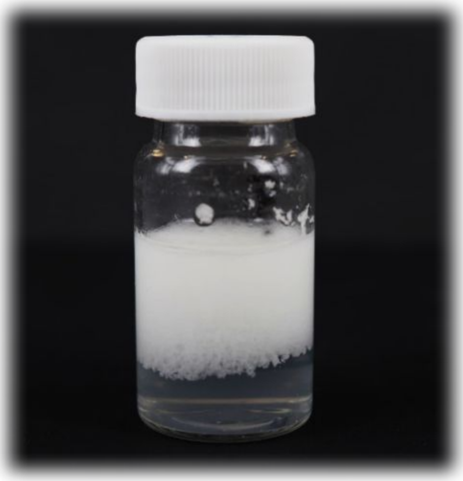

Figure S5. Oil-in-water emulsion in the presence of unmodified cellulose nanofibers.

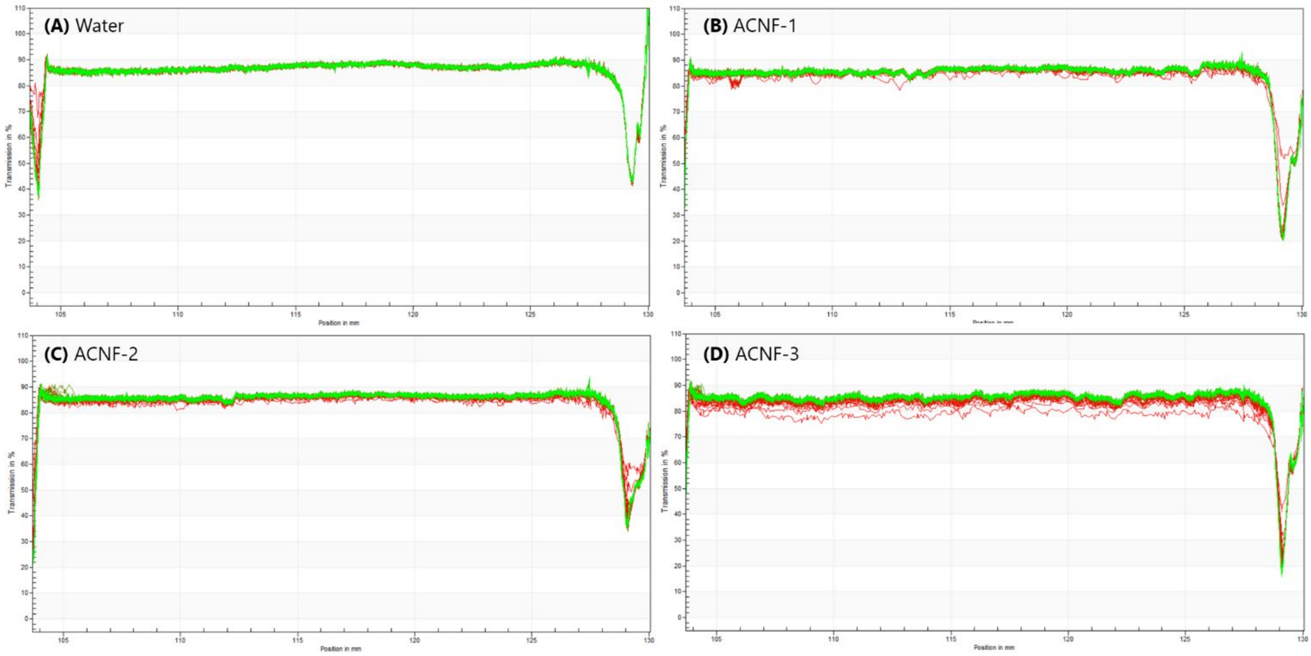

Figure S6. Light transmission (%) indicating the stability of (A) water and the aqueous dispersion of (B) ACNF-1, (C) ACNF-2, and (D) ACNF-3.

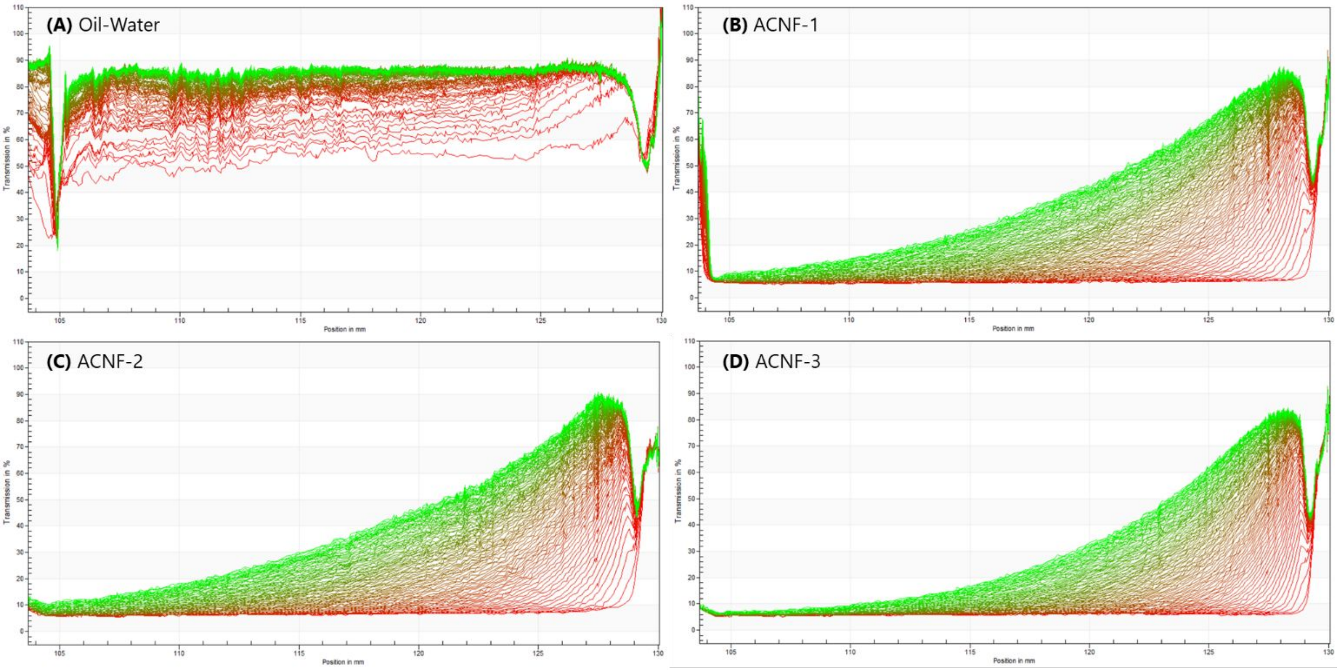

Figure S7. Light transmission (%) indicating the stability of (A) oil-water and the oil-water emulsions stabilized by (B) ACNF-1, (C) ACNF-2, and (D) ACNF-3.

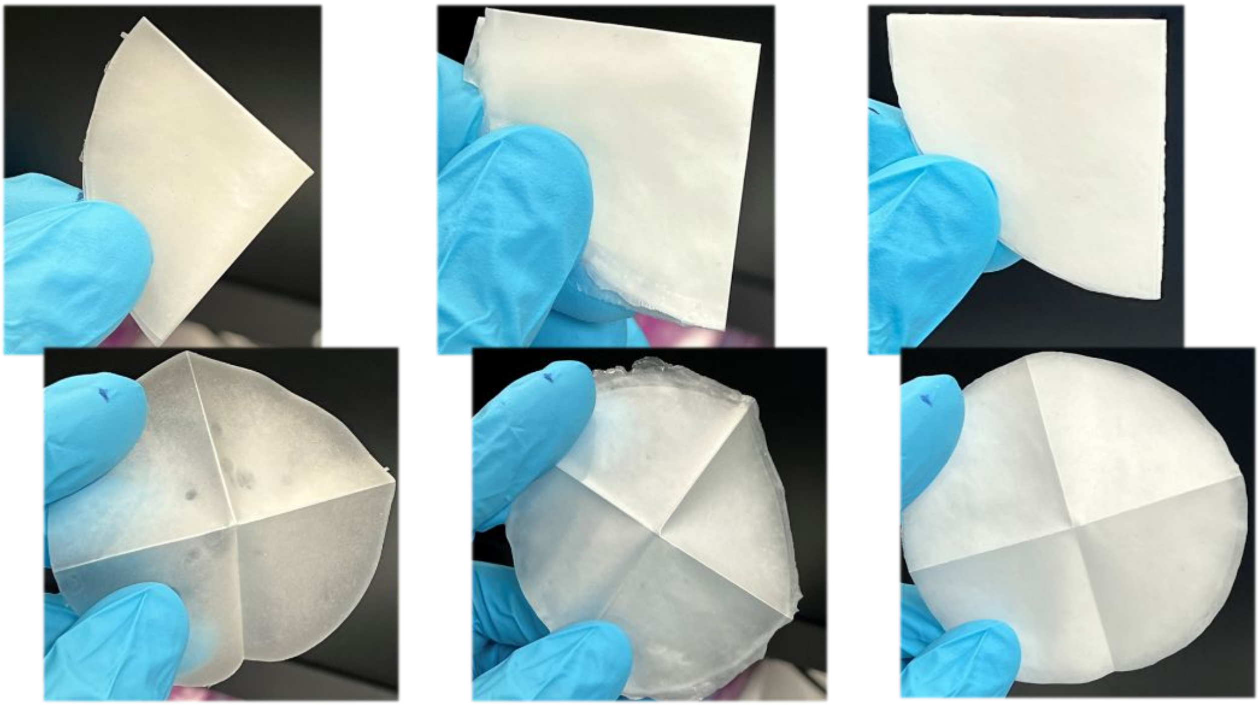

ACNF-1 ACNF-2 ACNF-3

Figure S8. Foldability of films of amphiphilic cellulose nanofibers (ACNFs).

Table S1. The water contact angle values of ACNF films (taken after every 15 seconds for 3 minutes).

Sr. No.	Water Contact Angle (°)		
	**ACNF-1**	**ACNF-2**	**ACNF-3**
1	105.2	110.8	116.3

2	104.6	109.9	116.1
3	103.6	107.1	115.6
4	103.3	105.0	115.4
5	102.6	108.4	114.9
6	101.8	99.3	114.5
7	100.9	98.9	113.8
8	100.7	98.5	113.8
9	100.3	97.9	113.2
10	99.9	97.3	113.1
11	98.7	96.9	112.7
12	98.4	96.4	112.3
13	97.6	95.3	112.1

Table S2. Tensile strength, tensile modulus, and elongation at break of films of amphiphilic cellulose nanofibers (ACNFs).

**Sr. No.**	**ACNF-1**			**ACNF-2**			**ACNF-3**		
	**TS (MPa)**	**TM (GPa)**	**EB (%)**	**TS (MPa)**	**TM (GPa)**	**EB (%)**	**TS (MPa)**	**TM (GPa)**	**EB (%)**
**1**	127.0	6.9	2.8	103.2	6.2	2.4	35.9	4.5	1.1
**2**	94.3	6.2	2.0	104.2	5.8	2.6	34.1	4.3	1.1
**3**	98.9	6.6	2.0	113.0	5.6	3.0	40.0	4.5	1.2
**4**	117.3	6.3	2.8	112.9	6.0	2.8	62.7	5.1	1.9
**5**	125.4	7.5	2.4	106.6	6.4	2.4	58.8	5.4	1.8
**6**	126.7	8.0	2.3	101.9	5.6	2.7	65.6	5.7	1.9
**Average**	114.9	6.9	2.4	107.0	5.9	2.7	49.5	4.9	1.5

Table S3. Names and references of materials are presented in Figure 5E.

**Sr. No.**	**Abbreviation**	**Full Name**	**Tensile Strength (MPa)**	**Reference**
**1**	CA	Cellulose acetate	95	[2]
**2**	CAP	Cellulose acetate butyrate	51	[2]
**3**	PP	Cellulose laureate	32	[2]
**4**	PS	Polystyrene	61	[2]
**5**	PCl	Poly(ε-caprolactone)	58	[2]
**6**	PET	Poly(ethylene terephthalate)	41.4	[2]
**7**	PHB	Poly(3-hydroxybutyrate)	62	[2]
**8**	LDPE	Low density polyethylene	31.8	[2]
**9**	HDPE	High density polyethylene	51	[2]
**10**	PLA	Polylactic acid	72	[2]
**11**	PVC	Polyvinyl chloride	68.9	[2]
**12**	ECNF	Esterified Cellulose Nanofibers	60	[3]

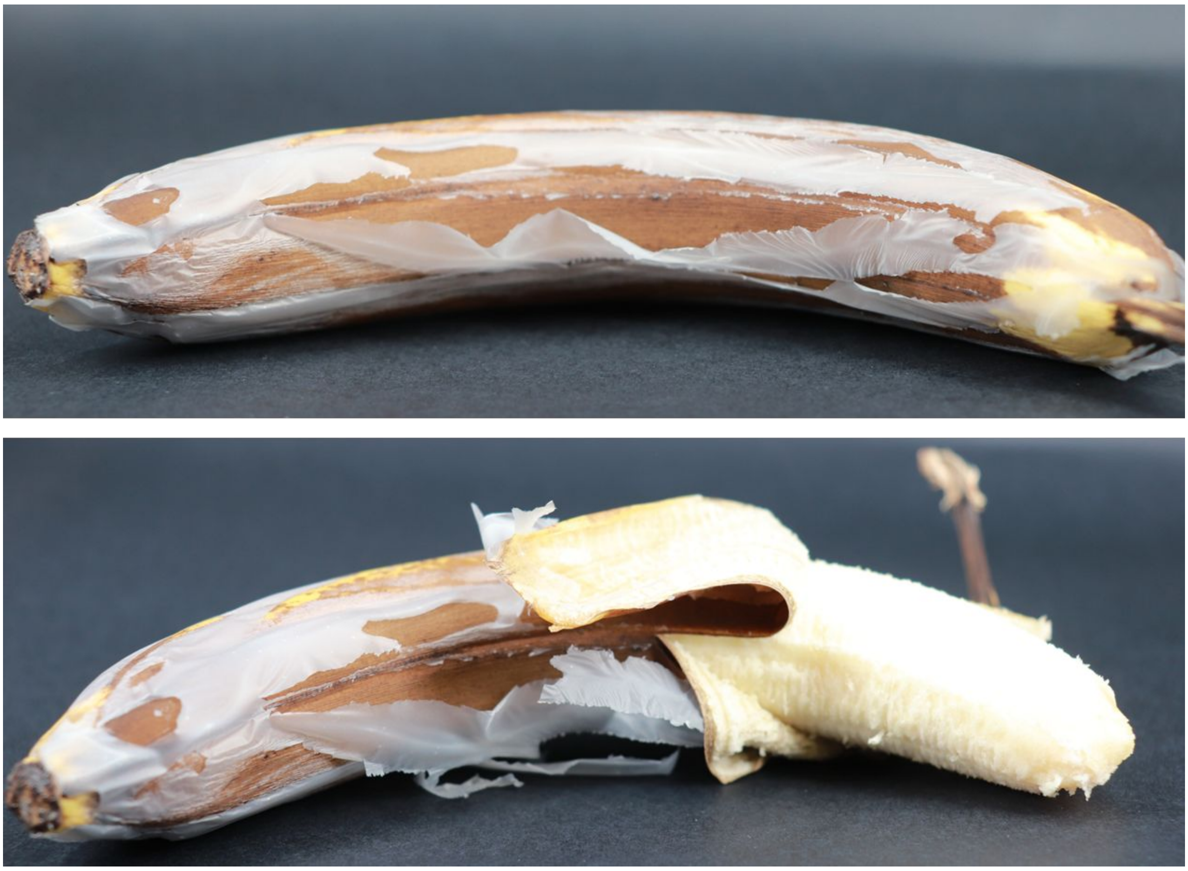

Figure S9. Banana coated with unmodified cellulose nanofibers and monitored for 6 days.

References

(1) Laitinen, O; Suopajärvi, T; Liimatainen, H. Enhancing Packaging Board Properties Using Micro- and Nanofibers Prepared from Recycled Board. *Cellulose 27*. https://doi.org/10.1007/s10570-020-03264-w.

(2) Wypych, G. *Handbook of Polymers*; 2022.

(3) Liu, S.; Zhang, Q.; Gou, S.; Zhang, L.; Wang, Z. Esterification of Cellulose Using Carboxylic Acid-Based Deep Eutectic Solvents to Produce High-Yield Cellulose Nanofibers. *Carbohydr. Polym.* **2021**, *251*. https://doi.org/10.1016/j.carbpol.2020.117018.
